# Sex differences in factors associated with neck pain among undergraduate healthcare students: a cross-sectional survey

**DOI:** 10.1186/s12891-022-05782-z

**Published:** 2022-09-03

**Authors:** Bi’e Zheng, Lifeng Zheng, Ming Li, Jianping Lin, Yuxiang Zhu, Liuzhisheng Jin, Roushi You, Yifang Gao, Xia Liu, Shizhong Wang

**Affiliations:** 1 Department of Rehabilitation, the First Affiliated Hospital of Fujian Medical University, 20 Chazhong Road, Fuzhou, Fujian China; 2 Department of Orthopedics, the First Affiliated Hospital of Fujian Medical University, 20 Chazhong Road, Fuzhou, Fujian China; 3 The School of Health, Fujian Medical University, 1 Xueyuan Road, University Town, Fuzhou, Fujian China

**Keywords:** Neck pain, Healthcare students, Sex differences, Poor posture, Psychological distress

## Abstract

**Background:**

Neck pain is widespread among students in healthcare-related fields. Although neck pain is more prevalent in females, since most research involves mixed-sex samples we know very little about sex differences in contributors to neck pain. Thus, this study sought to explore sex differences in the risk factors for neck pain in this high-risk population.

**Methods:**

This cross-sectional study was conducted in China in 2021 and included a sample of 1921 undergraduate healthcare students (693 males, 1228 females) from 7 health professional schools at Fujian Medical University. We collected data on neck pain symptoms, demographics, behavioral and psychological factors. Multiple regression analysis was conducted to examine sex differences in the risk factors of neck pain.

**Results:**

The overall prevalence of neck pain was 41.6% with female students having a higher prevalence than male students (44.4% vs. 36.7%, respectively). The adjusted analyses showed that self-study time ≥ 6 h/day (OR = 1.44, 95% CI:1.13-1.83), flexed neck posture >20 degrees (OR = 2.19, 95% CI: 1.28-3.74), static duration posture >2 h (OR = 1.42, 95% CI: 1.02-1.97), and psychological distress (high: OR = 2.04, 95% CI:1.42-2.94; very high: OR = 2.50, 95% CI:1.57-3.74; respectively) were independent factors for neck pain in females. Among males, self-study time ≥ 6 h/day (OR = 1.43, 95% CI: 1.02-2.01) and psychological distress (moderate: OR = 2.04, 95% CI:1.28-3.25; high: OR = 2.37, 95% CI:1.49-3.79; very high: OR = 2.97, 95% CI:1.75-5.02; respectively) were significant risk factors for neck pain.

**Conclusions:**

These findings suggest that the risk profiles of neck pain differ between females and males. The modifiable risk factors for neck pain, such as prolonged self-study time and elevated psychological distress, as well as poor posture among females, could be targeted through health promotion interventions in university settings.

## Introduction

Neck pain is a major health problem worldwide with an annual prevalence rates of 37.2% (range 16.7-75.1%) [1]. Healthcare professionals have developed various management and prevention measures for neck pain; paradoxically, neck pain is highly prevalent among healthcare professionals due to the nature of their work [2, 3]. However, it has been suggested that healthcare professionals experience neck pain even before they start working. An alarmingly high rate of musculoskeletal pain — especially neck pain — has been reported among undergraduate students studying to be healthcare professionals, with a reported annual prevalence ranging from 23.6 to 69.2% [4, 5]. Neck pain can pose a large burden to both individuals and society due to treatment costs, disruption of well-being, and loss of productivity [6, 7]. Thus, there is an urgent need to identify the risk factors of neck pain and develop effective interventions in this young, susceptible population.

The etiology of neck pain is complex and believed to be a biopsychosocial problem [1, 8]. Multiple risk factors for neck pain have been reported in previous studies, such as being female, an older age, a higher BMI, smoking, electronic devices overuse, awkward/sustained postures, physical inactivity, negative emotions, and lack of social support [1, 6, 8]. The global COVID-19 pandemic has posed huge changes to people’s daily routines, and healthcare students have been seriously affected. The pandemic and associated restrictions have negatively impacted their academic, lifestyle and mental health, which may precipitate or exacerbate neck pain [9–11]. However, studies of potential risk factors for neck pain are generally inconsistent, and evidence that can support the development of management strategies is limited [8, 12]. For example, some studies of healthcare students found that being female, an older age, and a higher BMI were not associated with neck pain [13–17]. Similarly, the findings regarding psychological factors associated with neck pain in healthcare students are mixed and thus inconclusive. Some studies report that psychological disorders are associated with an increased risk of neck pain, while others found no significant association [13–16].

Increasing studies have reported sex differences in lifestyle behaviors, and psychological status in healthcare students [18–21]. According to published reports, female students are less likely to be physically active than males and spend more time in sedentary activity [18, 19]. Additionally, under stressful conditions at medical universities, female students tend to suffer more from mental health problems (e.g. psychological distress, depression and anxiety) than their male counterparts [20, 21]. In this regard, some distinctions may exist in the association between neck pain and related factors. As most research on neck pain among healthcare students is based on combined analysis of mixed-sex samples, we know very little about sex differences in factors for neck pain. Sex differences in the prevalence of musculoskeletal pain, including neck pain among various population, has been reported in many studies, which is proposed to be due to sex differences in pain sensitivity and muscle anatomy and pain sensitivity [8, 22, 23]. Furthermore, some authors have noted that sex differences in behavioral and psychological risk factors can influence musculoskeletal pain, e.g., females report a higher likelihood of poor posture and higher rates of co-morbid psychological distress [24–26]. Such findings can help optimize individual prevention and treatment strategies for musculoskeletal pain since behavioral and psychological factors are potentially modifiable, unlike demographic characteristics.

To fill this research gap, the current study examined the occurrence of neck pain in relation to behavioral and psychological factors among healthcare students, with specific reference to sex specificity. We hypothesized that the factors associated with neck pain in this population may relate to sex differences.

## Methods

### Design and participants

This cross-sectional study was conducted at the largest medical university in Fujian Province, which is located in southeast China, during the period of December 14 to 29, 2021. A cluster sampling approach was adopted to draw a 30% sample of 7931 healthcare students at this university. The target population was full-time students enrolled in years 1-3 from the School of Basic Medical Sciences, School of Stomatology, School of Nursing, School of Public Health, School of Medical Technology and Engineering, School of Health, and School of Pharmacy. All students in the selected classes were encouraged to participate in this survey. The purpose of the survey and fact that their anonymity and confidentiality would be maintained were explained to the students by the investigators before obtaining their informed consent. Students completed the questionnaires via the online platform Wenjuanxing (www.wjx.cn). Most students took approximately 6-10 minutes to complete the questionnaire. The same IP address could be used only once to complete the questionnaire to prevent multiple responses from the same student. This study was approved by the Research Ethics Committee of Fujian Medical University (file reference:2021-159).

### Sample size

The sample size of the study was calculated by the formula: n = (z_a/2_)^2^ p (1 − p) /d2, where, *n* = sample size, z_a/2_ = 1.96, p = a previous rate of 29.2% proportion prevalence of neck pain among Chinese undergraduate students, and d = margin of error (3%), which produced a sample size of 883 [27]. Since a cluster sampling strategy was adopted with a design effect of 2, the final sample size estimated was 2355 subjects accounting for a 25% non-response rate.

### Neck pain

Neck pain was assessed using the Nordic Musculoskeletal Questionnaire [28]. This questionnaire was validated and adapted for various population [4, 15, 29]. Specifically, participants were asked the following question: “During the past 12 months, have you at any time had ache, pain or discomfort in the neck?” A body map was included to allow students to report neck pain by referencing specific neck region [28, 29]. The response options were “no” or “yes”, with “yes” denoting relevant pain in the neck region.

### Demographic, behavioral, and psychological questionnaires

Demographic characteristics, including age, gender (male or female), year of study (first year, second year, or third year), weight, and height, were collected from all participants using a questionnaire. Body mass index (BMI) was calculated as weight (kg)/height (meter)^2^ and then categorized as underweight (< 18.5), normal (18.5-24.9), overweight (25.0-29.9), and obese (> 30).

The following behaviors were assessed: smoking status (never, ex-smoker, or current smoker), computer usage (<2 h/day, or ≥ 2 h/day), smartphone usage (<6 h/day, or ≥ 6 h/day), self-study time (<6 h/day, or ≥ 6 h/day), sitting habitual neck posture (upright, slightly flexed 10-20 degrees, or sharply flexed >20 degrees; with a reference picture about sagittal neck posture), static duration posture (<1 h, 1-2 h, or >2 h), and leisure time physical activity (occasionally; regularly, 1-2 times/week, or regularly, ≥3 times/week; 1 time means continuous physical activity for at least 30 min per week, and feeling warm or sweaty) [10, 15–17, 30].

Psychological factors were assessed using the Kessler Psychological Distress Scale (K10). We used the K10 because it is an internationally validated, brief screening instrument to assess non-specific psychological symptoms in population-based epidemiological studies, with a focus on depression and anxiety [31]. Participants rated the 10 items on a 5-point Likert scale (ranging from none of the time to all the time). Scores range from 10 to 50, with a higher score indicating more psychological distress. The interpretation of K10 scores is based on a four-level categorization: low (10-15), moderate (16-21), high (22-29), and very high (30-50) [32]. The Cronbach’s alpha of the K10 in this sample was 0.925.

### Statistical analysis

Statistical analysis was performed using the IBM Statistical Package for Social Science program, version 25.0. Categorical variables are presented as numbers and percentages. The chi-square test was used to compare categorical variables between groups with and without neck pain. Binary logistic regression analyses were conducted separately for male and female students to identify sex-specific risk factors associated with neck pain. Univariate logistic regression models were used to identify potential confounders. Variables with *p* < 0.1 in the univariate logistic regression were selected for multiple logistic regression model. The results are presented as crude odds ratios (cORs), adjusted ORs (aORs), and 95% confidential intervals (CIs). A *p*-value < 0.05 was considered to be statistically significant.

## Results

A total of 2003 healthcare students completed the survey. Of these, 82 were excluded from analysis due to an overly short response duration (< 3 min) or errors in logic, resulting in a final sample of 1921 participants (a valid response rate = 95.9%). The characteristics of these participants are shown in Table 1. Participants’ ages ranged from 17 to 23 years with a median of 19 years; 37.1% were first year students, 31.3% were second year students, and 31.5% were third year students. The male: female sex ratio was approximately 1:2 (males: 36.1%, females: 63.9%), which may result from the sex imbalance at many Chinese colleges and universities [33]. The annual prevalence of neck pain was 41.6% (95% CI: 39.4-43.8) with female students having a higher prevalence than male students, 44.4% (95% CI: 41.6-47.2) and 36.7% (95% CI: 33.1-40.2), respectively.

**Table 1:** Baseline characteristics of all participants in this study (*n* = 1921)

	Total	Male *n* = 693 n (%)	Female *n* = 1228 n (%)	***p value***
Neck pain				**0.001**
No	1122 (58.4)	439 (63.3)	683 (55.6)	
Yes	799 (41.6)	254 (36.7)	545 (44.4)	
**Demographic**				
Age groups				0.179
17-19 years	1106 (57.6)	385 (55.6)	721(58.7)	
20-23 years	815 (42.4)	308 (44.4)	507 (41.3)	
BMI				**<0.001**
Underweight	333 (17.6)	86 (12.4)	247 (20.1)	
Normal	1319 (69.2)	457 (65.9)	872 (71.0)	
Overweight/Obese	259 (13.7)	150 (21.6)	109 (8.9)	
Year of study				0.070
First year	713 (37.1)	258 (37.2)	455 (37.1)	
Second year	602 (31.3)	236 (34.1)	366 (29.8)	
Third year	606 (31.5)	199 (28.7)	407 (33.1)	
**Behavior-related factors**				
Smoking status				**<0.001**
Never	1864 (97.0)	650 (93.8)	1214 (98.9)	
Ex-smoker	26 (1.4)	18 (2.6)	8 (0.7)	
Current smoker	31 (1.6)	25 (3.6)	6 (0.5)	
Computer usage				**<0.001**
<2 h/day	1051 (54.7)	307 (44.3)	744 (60.6)	
≥ 2 h/day	870 (45.3)	386 (55.7)	484 (39.4)	
Smartphone usage				**<0.001**
<6 h/day	436 (22.7)	186 (26.8)	250 (20.4)	
≥ 6 h/day	1485 (77.3)	507 (73.2)	978 (79.6)	
Self-study time				**0.018**
<6 h/day	1254 (65.3)	476 (68.7)	778 (63.4)	
≥ 6 h/day	667 (34.7)	217 (31.3)	450 (36.6)	
Sitting habitual neck posture				**<0.001**
Upright	558 (29.0)	224 (32.3)	334 (27.2)	
Slightly flexed (10-20 degrees)	884 (46.0)	300 (43.3)	584 (47.6)	
Sharp flexed (>20 degrees)	479 (24.9)	169 (24.4)	310 (25.2)	
Static duration posture				**<0.001**
<1 h	157 (8.2)	82 (11.8)	75 (6.1)	
1-2 h	1070 (55.7)	416 (46.0)	654 (53.3)	
>2 h	694 (36.1)	195 (28.1)	499 (40.6)	
Leisure time physical activity				**<0.001**
Occasionally	1267 (66.0)	322 (46.5)	945 (77.0)	
Regularly, 1-2 times/week	318 (16.6)	147 (21.2)	171 (13.9)	
Regularly, ≥3 times/week	336 (17.5)	224 (32.3)	112 (9.1)	
**Psychological factor**				
Psychological distress				**<0.001**
Low (1-15)	361 (18.8)	167 (24.1)	194 (15.8)	
Moderate (16-21)	584 (30.4)	212 (30.6)	372 (30.3)	
High (22-29)	614 (32.0)	199 (28.7)	415 (33.8)	
Very high (30-50)	362 (18.8)	115 (16.6)	247 (20.1)	

*BMI* body mass index

There were statistically significant sex differences in the demographics, behavioral and psychological factors. Compared female respondents, a greater proportion of male respondents reported being “overweight/obese” and a “current smoker”. Significantly more males than females reported high computer usage time per day, whereas significantly more females than males reported high smartphone usage time per day (all *p* < 0.001). Furthermore, prolonged self-study time, a sitting flexed neck posture, prolonged static posture, physical inactivity, and psychological distress were reported more often by female students (all *p* < 0.05).

Table 2 shows that male students with neck pain were more likely to report a prolonged self-study time, a low level of physical activity, and an elevated level of psychological distress (all *p* < 0.05). The other variables did not differ significantly between males with and without neck pain. In the univariate analyses, self-study time (≥6 h/day: cOR = 1.47, *p* = 0.022) and psychological distress (moderate: cOR = 2.09, high: cOR = 2.51, very high: cOR = 3.22; all *p* < 0.01) were associated with increased neck pain.

**Table 2:** Univariate analysis of risk factors for neck pain in male students (*n* = 693)

	Without neck pain *n* = 439, n (%)	With neck pain *n* = 254, n (%)	***p value***	cOR (95% CI)	***p value***
**Demographic**					
Age groups			0.078		
17-19 years	255 (58.1)	130 (51.2)		Ref.	
20-23 years	184 (41.9)	124 (48.8)		1.32 (0.97-1.80)	0.078
BMI			0.929		
Underweight	56 (12.8)	30 (11.8)		0.92 (0.57-1.49)	0.740
Normal	289 (65.8)	168 (66.1)		Ref.	
Overweight/Obese	94 (21.4)	56 (22.0)			
Year of study			0.853		
First year	166 (37.8)	92(36.2)		Ref.	
Second year	150 (34.2)	86(33.9)		1.03 (0.72-1.49)	0.857
Third year	123 (28.0)	76(29.9)		1.12 (0.76-1.64)	0.578
**Behavior-related factors**					
Smoking status			0.725		
Never	413 (94.1)	237 (93.3)		Ref.	
Ex-smoker	12 (2.7)	6 (2.4)		0.87 (0.32-2.35)	0.786
Current smoker	14 (3.2)	11 (4.3)		1.37 (0.61-3.07)	0.445
Computer usage			0.934		
<2 h/day	195 (44.4)	112 (44.1)		Ref	
≥ 2 h/day	244 (55.6)	142 (55.9)		1.01 (0.74-1.38)	0.934
Smartphone usage			0.975		
<6 h/day	118 (26.9)	68 (26.8)		Ref.	
≥ 6 h/day	321 (73.1)	186 (73.2)		1.01 (0.71-1.43)	0.975
Self-study time			**0.022**		
<6 h/day	315 (71.8)	161 (63.4)		Ref.	
≥ 6 h/day	124 (28.2)	93 (36.3)		**1.47 (1.06-2.04)**	**0.022**
Sitting habitual neck posture			0.334		
Upright	58 (13.2)	24 (9.4)		Ref.	
Slightly flexed (10-20 degrees)	260 (59.2)	156 (61.4)		1.45 (0.87-2.43)	0.158
Sharp flexed (>20 degrees)	121(27.6)	74 (29.1)		1.48 (0.85-2.58)	0.169
Static duration posture			0.486		
<1 h	149 (33.9)	75 (29.5)		Ref.	
1-2 h	186 (42.4)	114 (44.9)		1.22 (0.85-1.75)	0.287
>2 h	104 (23.7)	65 (25.6)		1.24 (0.82-1.88)	
Leisure time physical activity			**0.030**		
Occasionally	200 (45.6)	122 (48.0)		Ref.	
Regularly, 1-2 times/week	83 (18.9)	64 (25.2)		1.26 (0.85-1.88)	0.246
Regularly, ≥3 times/week	156 (35.5)	68 (26.8)		0.72 (0.50-1.03)	0.070
**Psychological factor**					
Psychological distress			**<0.001**		
Low (1-15)	130 (29.6)	37 (14.6)		Ref.	
Moderate (16-21)	133 (30.3)	79 (31.1)		**2.09 (1.32-3.30)**	**0.002**
High (22-29)	116 (26.4)	83 (32.7)		**2.51 (1.59-3.99)**	**<0.001**
Very high (30-50)	60 (13.7)	55 (21.7)		**3.22 (1.92-5.40)**	**<0.001**

*Ref*  Reference, *BMI* body mass index, c*OR* crude odds ratio, 95%*CI c*onfidence interval

Table 3 shows that female students with neck pain tended to have a prolonged self-study time, flexed neck posture, prolonged static posture, and elevated level of psychological distress compared with those without neck pain (all *p* < 0.05). Univariate analysis identified longer self-study time (≥ 6 h/day: cOR = 1.39, *p* = 0.006), sitting habitual neck posture (sharply flexed >20 degrees: cOR = 2.38, *p* = 0.001), static duration of posture (>2 h: cOR = 1.68, *p* = 0.001), and psychological distress (high: cOR = 2.19, very high: cOR = 2.81, respectively; all *p* < 0.001) as potential risk factors for neck pain.

**Table 3:** Univariate analysis of risk factors for neck pain in female students (*n* = 1228)

	Without neck pain *n* = 683, n (%)	With neck pain *n* = 545, n (%)	***p value***	cOR (95% CI)	***p value***
**Demographic**					
Age groups			0.817		
17-19 years	403 (59.0)	318 (58.3)		Ref.	
20-23 years	280 (41.0)	227 (41.7)		1.03 (0.82-1.29)	0.817
BMI			0.368		
Underweight	147 (21.5)	100 (18.3)		0.83 (0.62-1.10)	0.190
Normal	478 (70.0)	394 (72.3)		Ref.	
Overweight/Obese	58 (8.5)	51 (9.4)		1.07 (0.72-1.59)	0.751
Year of study			0.848		
First year	257 (37.6)	198 (36.3)		Ref.	
Second year	204 (29.9)	162 (29.7)		1.03 (0.78-1.36)	0.830
Third year	222 (32.5)	222 (33.9)		1.08 (0.83-1.42)	0.568
**Behavior-related factors**					
Smoking status			0.843		
Never	675 (98.8)	543 (98.9)		Ref.	
Ex-smoker	4 (0.6)	4 (0.7)		1.25 (0.31-5.03)	0.751
Current smoker	4 (0.6)	2 (0.4)		0.59 (0.11-3.43)	0.588
Computer usage			0.231		
<2 h/day	424 (62.1)	320 (58.7)		Ref.	
≥ 2 h/day	259 (37.9)	225 (41.3)		1.15 (0.91-1.45)	0.231
Smartphone usage					
<6 h/day	150 (22.0)	68 (18.3)		Ref.	
≥ 6 h/day	533 (78.0)	186 (81.7)		1.25 (0.94-1.66)	0.119
Self-study time			**0.008**		
<6 h/day	456 (66.8)	322 (59.1)		Ref.	
≥ 6 h/day	227 (33.2)	223 (40.9)		**1.39 (1.10-1.76)**	**0.006**
Sitting habitual neck posture			**<0.001**		
Upright	52 (7.6)	23 (4.2)		Ref.	
Slightly flexed (10-20 degrees)	388 (56.8)	266 (48.8)		1.55 (0.93-2.59)	0.095
Sharp flexed (>20 degrees)	243 (35.6)	256 (47.0)		**2.38 (1.41-4.01)**	**0.001**
Static duration posture			**0.003**		
<1 h	201 (29.4)	133 (24.4)		Ref.	
1-2 h	335 (49.0)	249 (45.7)		1.12 (0.85-1.48)	0.405
>2 h	147 (21.5)	163 (29.9)		**1.68 (1.23-2.29)**	**0.001**
Leisure time physical activity			0.555		
Occasionally	533 (78.0)	412 (75.6)		Ref.	
Regularly, 1-2 times/week	89 (13.0)	82 (15.0)		1.19 (0.86-1.65)	0.292
Regularly, ≥3 times/week	61 (8.9)	51 (9.4)		1.08 (0.73-1.60)	0.696
**Psychological factor**					
Psychological distress			**<0.001**		
Low (1-15)	133 (19.5)	61 (11.2)		Ref.	
Moderate (16-21)	235 (34.4)	137 (25.1)		1.27 (0.88-1.83)	0.203
High (22-29)	207 (30.3)	208 (38.2)		**2.19 (1.53-3.14)**	**<0.001**
Very high (30-50)	109 (15.8)	139 (25.5)		**2.81 (1.89-4.16)**	**<0.001**

*Ref* Reference, *BMI* body mass index, c*OR* crude odds ratio, 95% *CI *confidence interval

Table 4 indicates that a self-study time ≥ 6 h/day was significantly associated with neck pain in both males and females (aOR = 1.43 and aOR = 1.44, respectively; all *p *<0.05). Notably, males with very high, high, and moderate psychological distress had a significantly increased risk of neck pain (aOR = 2.04, aOR = 2.37 and aOR = 2.97, respectively; all *p*<0.01). A similar association was observed in females, with the odds of neck pain increasing with high or very high psychological distress (aOR = 2.04 and aOR = 2.50, respectively; all *p *<0.001). Furthermore, a sitting habitual neck posture (sharply flexed >20 degrees: aOR = 2.19, *p* = 0.004), and static duration of posture were significantly associated with neck pain in female students (>2 h: aOR = 1.42, *p* = 0.036). However, there was no such relationship in male students.

**Table 4:** Multivariate analysis of risk factors for neck pain separated by sex (*n* = 1921)

	Males		Females	
	aOR (95% CI)	***p value***	aOR (95% CI)	***p value***
**Demographic**				
Age groups				
17-19 years	Ref.		-	-
20-23 years	1.19 (0.86-1.63)	0.302	-	-
**Behavior-related factors**				
Self-study time				
<6 h/day	Ref.		Ref.	
≥ 6 h/day	**1.43 (1.02-2.01)**	**0.039**	**1.44 (1.13-1.83)**	**0.003**
Sitting habitual neck posture				
Upright	-	-	Ref.	
Slightly flexed (10-20 degrees)	-	-	1.57 (0.93-2.66)	0.095
Sharp flexed (>20 degrees)	-	-	**2.19 (1.28-3.74)**	**0.004**
Static duration posture				
<1 h	-	-	Ref.	
1-2 h	-	-	1.04 (0.78-1.37)	0.812
>2 h	-	-	**1.42 (1.02-1.97)**	**0.036**
Leisure time physical activity				
Occasionally	Ref.		-	-
Regularly, 1-2 times/week	1.23 (0.82-1.86)	0.313	-	-
Regularly, ≥3 times/week	0.80 (0.55-1.16)	0.238	-	-
**Psychological factor**				
Psychological distress				
Low (1-15)	Ref.		Ref.	
Moderate (16-21)	**2.04 (1.28-3.25)**	**0.003**	1.18 (0.81-1.72)	0.379
High (22-29)	**2.37 (1.49-3.79)**	**< 0.001**	**2.04 (1.42-2.94)**	**<0.001**
Very high (30-50)	**2.97 (1.75-5.02)**	**< 0.001**	**2.50 (1.57-3.74)**	**<0.001**

*Ref* Reference, *BMI* body mass index, a*OR* adjusted odds ratio, 95% *CI* confidence interval

## Discussion

In current study, we found that a significant proportion of undergraduate healthcare students in China experienced neck pain in the previous 12 months, with female students having a higher prevalence than male students. Sex differences in the prevalence of neck pain among healthcare students have been reported in other studies [5, 14, 19]. We further identified sex-specific risk profiles for neck pain in this high-risk population, which supports our research hypothesis. Specifically, among female students, self-study time, habitual flexed neck posture, static duration of posture, and psychological distress were significantly associated with neck pain. In contrast, only self-study time and psychological distress were significantly associated with neck pain in the adjusted model for male students. To the best of our knowledge, this is the first study to explore sex different in risk factors with neck pain among healthcare students. Accordingly, some meaningful implications can be drawn from our findings.

The association between time spent on electronic devices and neck pain is debatable [12, 34]. According to a prior study, medical students with smartphone usage >6 h/day were 2.782 times more likely to report neck pain [17]. Among our participants, females tended to show greater use of smartphones, while males spent more time on computers than females. Our results are consistent with other research of university students reporting sex differences in the time spent on electronic devices [33]. However, no association was found with either daily duration of smartphone or computer use with neck pain in each sex. This may be partly because the data in our study were self-reported, which could have led to bias. This finding is, however, similar to that of a recent study reporting no significant association between increased time spent on electronic devices and musculoskeletal pain in medical students in Jordan during remote learning [11]. Furthermore, our results show no association of older age, year of study, BMI, and smoking status with neck pain in each sex. Future research is thus required to examine these associations.

Healthcare students frequently engage in studying activities because of their demanding academic load. Our results showed an independent association between prolonged studying and neck pain in males and females; specifically, students who spent ≥6 h/day in self-study were more likely to experience neck pain. Previous studies have reported that poor posture has a significant effect on neck pain among healthcare students [4, 16]. In particular, poorer posture was associated with a higher prevalence of neck pain while participating in remote learning during the pandemic [11]. In the present study, a habitual neck posture and static duration of posture apparently differed between males and females. Further, we found that a sitting habitual neck posture and static duration of posture were significantly associated with neck pain in females. However, there was no such relationship in males. Based on our findings, females with flexed neck posture (>20 degrees) and static posture over 2 hours had an increased risk of neck pain 2.19 times and 1.42 times, respectively. Previous studies have demonstrated that non-neutral postures contribute to cumulative biomechanical load on the cervical spine, while sustained postural loading affects these structures (e.g., strain muscles and ligaments, compress nerves) and can become a cause of neck pain [35, 36]. The present findings extend the literature regarding the association of poor posture and neck pain being female-specific, which could partially explain the higher prevalence of neck pain in females. A prospective study by Richards et al. also supports posture as a sex-specific risk factor; however, they found that females in late adolescence who sat with an upright sitting posture had a greater risk of persistent neck pain as young adults than those with a thorax/forward head or intermediate posture [37]. More research is thus warranted to determine the optimal posture to prevent and treat neck pain.

Generally, regular physical activity has benefits for overall physical and mental health. Based on leisure time physical activity reported by our participants, almost two thirds had irregular physical activity and females were less physically active than males. This is consistent with findings from other studies showing sex differences in physical activity levels [18, 19, 38]. Contrary to the hypothesis, our results showed that physical activity was not associated with neck pain in each sex. The results of this study are thus discordant with those of prior studies reporting that healthcare students with regular physical activity have a reduced occurrence of neck pain compared with those who were physical inactive [5, 16, 17]. A possible explanation for this discrepancy might be that we collected data during the pandemic, and the various restrictions may have dissuaded students from engaging in the recommended levels of physical activity. However, the evidence for the role of physical activity in neck pain in the existing literature has been mixed, with some studies suggesting regular physical activity can be beneficial while others indicate no effect or even harmful effects [10, 12, 39, 40]. From a public health perspective, specific types and intensities of physical activity could be developed and promoted to help prevent neck pain.

Psychological distress among healthcare students has become a global concern — especially during the pandemic [9, 20, 21]. In this study, psychological distress was found to be more prevalent in females compared to males, which is consistent with sex differences in the prevalence of psychological distress in this population [20, 21]. Further, our study showed that psychological distress was positively associated with neck pain in both males and females; specifically, those with higher levels of psychological distress were more likely to experience neck pain. Our results are compatible with other studies that investigated the association between psychological distress and neck pain, despite the different assessment scales for psychological distress [41–43]. Some prospective studies have suggested that psychological distress as a meaningful predictor of the development and persistence in neck pain [26, 44, 45]. To our knowledge, few studies prior to this work have examined this association among healthcare students, despite the high prevalence of psychological distress and neck pain in this population. Due to increased recognition of the important role of psychological factors in neck pain, psychological interventions are increasingly being used in the management of neck pain [46–48]. It is also important to note that psychological distress was more strongly associated with neck pain in males than in females, as shown in our study, although females reported higher levels of psychological distress than males. This finding is supported by several studies in adolescents and the general population showing that males report a higher association with musculoskeletal pain-related emotions (e.g., depression and catastrophizing) than females [49, 50]. However, it is unclear how sex influences the underlying mechanism of this observation. Nevertheless, these findings suggest that raising awareness among healthcare providers about psychological interventions for males is recommended to prevent males with neck pain from being treated less aggressively than females.

### Limitations and strengths

Some limitations of this study need to be noted. Firstly, as we did not differentiate between acute and chronic neck pain, the findings need to be interpreted with caution. Secondly, as the data were collected using a self-report questionnaire, there may be discrepancies between what the students reported and their actual experiences. Thirdly, our cross-sectional design prevents drawing causal inferences from the associations. Future prospective studies are required to confirm our results. Fourthly, other neck pain-related factors were not accounted for, such as medication use, past pain experience, socioeconomic status, and social support. Thus, generalization is limited. Finally, since recruitment was restricted to undergraduate students at one medical university, our sample might not be representative of all Chinese healthcare students and students from other countries.

The strengths of the current study are its relatively large sample, which allowed us to identify sex differences in several factors associated with neck pain in healthcare students and offer potential implications for healthcare providers. Of further concern is the fact that neck pain during this period of life may adversely affect the long-term health of healthcare students. It is thus imperative to increase awareness among healthcare students during medical education about risk factors for neck pain and encourages healthy lifestyle habits, especially informing female students of proper posture. On the other hand, our study highlights the importance of accounting for psychological components: identifying and addressing psychological distress in healthcare students may be an effective intervention to prevent and control neck pain. Lastly, additional research is needed to obtain a better understanding of the mechanisms underlying the sex differences in these associations.

## Conclusions

In conclusion, our findings indicated that prolonged self-study time and elevated psychological distress were significantly associated with neck pain in both sexes. In addition, poor posture in females is likely to increase the risk of neck pain. Early management of neck pain based on sex-related differences in risk factors should be considered in university settings to minimize the burden of neck pain among future healthcare professionals.

## Data Availability

The data presented in this study are available on request from the corresponding author. The data are not publicly available due to privacy reasons.

## Abbreviations

BMI: Body mass index
Ref.: Reference
OR: Odds ratio
CI: Confidence interval
cOR: Crude odds ratio
aOR: Adjusted odds ratio
